# Immunogenicity of Mycobacterial Extracellular Vesicles Isolated From Host-Related Conditions Informs About Tuberculosis Disease Status

**DOI:** 10.3389/fmicb.2022.907296

**Published:** 2022-06-22

**Authors:** Sebastian Schirmer, Lucas Rauh, Sogol Alebouyeh, Mario Delgado-Velandia, Vivian C. Salgueiro, Laura Lerma, José L. Serrano-Mestre, Mikel Azkargorta, Félix Elortza, José L. Lavín, Maria Jesus García, María Teresa Tórtola Fernández, Susanne Gola, Rafael Prados-Rosales

**Affiliations:** ^1^Department of Medical Engineering and Biotechnology, Ernst-Abbe-Hochschule, University of Applied Sciences, Jena, Germany; ^2^Department of Preventive Medicine, Public Health and Microbiology, School of Medicine, Universidad Autónoma de Madrid, Madrid, Spain; ^3^CIBERESP (CIBER of Epidemiology and Public Health), Madrid, Spain; ^4^Proteomics Platform, CIC bioGUNE, Basque Research and Technology Alliance (BRTA), Derio, Spain; ^5^Bioinformatics Unit, Applied Mathematics Department, NEIKER-Basque Institute for Agricultural Research and Development, Basque Research and Technology Alliance (BRTA), Derio, Spain; ^6^Department of Genetics and Microbiology, Universitat Autònoma de Barcelona, Barcelona, Spain

**Keywords:** *Mycobacterium tuberculosis*, extracellular vesicles, immunogenicity, diagnostic biomarkers, serology, iron starvation

## Abstract

Tuberculosis (TB) still represents a major global health problem affecting over 10 million people worldwide. The gold-standard procedures for TB diagnosis are culture and nucleic acid amplification techniques. In this context, both lipoarabinomannan (LAM) urine test and rapid molecular tests have been major game changers. However, the low sensitivity of the former and the cost and the prohibitive infrastructure requirements to scale-up in endemic regions of the latter, make the improvement of the TB diagnostic landscape a priority. Most forms of life produce extracellular vesicles (EVs), including bacteria despite differences in bacterial cell envelope architecture. We demonstrated that *Mycobacterium tuberculosis* (*Mtb*), the causative agent of TB, produces EVs *in vitro* and *in vivo* as part of a sophisticated mechanism to manipulate host cellular physiology and to evade the host immune system. In a previous serology study, we showed that the recognition of several mycobacterial extracellular vesicles (MEV) associated proteins could have diagnostic properties. In this study, we pursued to expand the capabilities of MEVs in the context of TB diagnostics by analyzing the composition of MEVs isolated from *Mtb* cultures submitted to iron starvation and, testing their immunogenicity against a new cohort of serum samples derived from TB+ patients, latent TB-infected (LTBI) patients and healthy donors. We found that despite the stringent condition imposed by iron starvation, *Mtb* reduces the number of MEV associated proteins relative to iron sufficient conditions. In addition, TB serology revealed three new MEV antigens with specific biomarker capacity. These results suggest the feasibility of developing a point-of-care (POC) device based on selected MEV-associated proteins.

## Introduction

Tuberculosis (TB) is the leading cause of death due to a single bacterial infectious agent worldwide, with approximately 1.4 million deceased in 2020 (WHO, 2020b). Thus, TB remains one of the greatest global health threats. At present, there is an urgent need for TB biomarkers, which dampens the advance in highly demanding approaches such as vaccine development or diagnostics. The gold-standard procedures for TB diagnosis are culture and nucleic acid amplification techniques. However, culture-based methods need laboratory infrastructure, trained personnel, and entail incubation times that considerably delay the outcome of the diagnosis. Although the World Health Organization (WHO) endorsed GeneXpert Mtb/RIF, among others, to improve the rapid diagnosis of drug-sensitive and resistant TB, it is costly and requires technological investment. Therefore, albeit being limited by reduced sensitivity of around 50% (Steingart et al., 2006), culture methods remain the standard approach for TB diagnosis in resource-limited settings. The rapid diagnosis of the disease is critical to reduce transmission, morbidity, and mortality. In this context, the WHO proposed a set of target product profiles (TPPs) (WHO, 2020a) to guide and encourage the development of point-of-care (POC) devices to enhance TB case detection. Consequently, TB biomarkers that can set the bases for the development of novel POC tests are urgently needed. One such TPP is a non–sputum biomarker test to start TB treatment at the time of the initial clinical visit (WHO, 2020a). Among these approaches is the detection of the mycobacterial cell wall-associated lipoglycan lipoarabinomannan (LAM) in urine. However, due to its moderate sensitivity, it is only recommended in HIV co-infected individuals with low CD4 cell counts (Shah et al., 2016). Recent versions of this method improved sensitivity, but this parameter is still far below that of culture- and molecular-based methods (reviewed in Flores et al., 2021).

Most forms of life produce extracellular vesicles (EVs). Since the first detection of bacterial EVs more than 60 years ago in *Escherichia coli* (Bishop and Work, 1965), subsequent studies have demonstrated their functional commonality despite differences in bacterial cell envelope architecture. Bacterial EVs are “spherical, membranous vesicles from microbial cell surfaces ranging in size from 20 to 500 nm in diameter” (Brown et al., 2015; Schwechheimer and Kuehn, 2015). We demonstrated that *Mycobacterium tuberculosis* (*Mtb*), the causative agent of TB, produces EVs *in vitro* and *in vivo* as part of a sophisticated mechanism to manipulate host cellular physiology and evade the host immune system (Prados-Rosales et al., 2011). *Mtb* EVs (MEVs) have immunomodulatory properties *in vitro* and when administered to mice (Prados-Rosales et al., 2011; Athman et al., 2015), they pose promising vaccine properties (Prados-Rosales et al., 2014a) and seem to be genetically regulated (Rath et al., 2013; White et al., 2018). Consequently, MEVs have generated considerable interest for their potential role in TB pathogenesis and their implications in the development of new preventive and therapeutic antitubercular strategies. Moreover, we reported that iron starvation, a host condition that drives the pathogen into a mode of slow proliferation, stimulates vesiculation in *Mtb* (Prados-Rosales et al., 2014b).

In the context of infection, host-derived EVs (HEVs), such as exosomes or microparticles, may remotely interfere with the immune system from the primary site of infection (Choi et al., 2015). Exosomes from macrophages infected with *Mtb* activate proinflammatory responses, drive dendritic cell maturation, and regulate antigen presentation, consistent with the exosome-associated bacterial cargo (Bhatnagar and Schorey, 2007). A major premise for antigen export from infected phagocytes is that *Mtb*-related antigens associate with host membranes and traffic into exosomes. However, reports exploring the origin of EVs in *Mtb*-infected macrophages challenged this view by showing that HEVs and MEVs may represent two independent populations of vesicles (Athman et al., 2015), indicating that the examination of just HEVs in the context of infection will provide only a partial picture of the interplay between *Mtb* and the host (Palacios et al., 2021). Since most of *Mtb*’s biology occurs inside of immune cells, EVs derived from this interaction can provide valuable information about the outcome of such interaction. Therefore, we hypothesize that deciphering the heterogeneity and immunogenicity of vesicles in the context of human *Mtb*-infection could have the potential to contribute to the development of the highly demanded approaches to tackle the disease. We have previously evaluated the serological responses to MEVs isolated from *Mtb* and BCG grown in high iron conditions in human TB individuals and controls showing that a set of three MEV-associated proteins produced a TB-specific serologic profile (Ziegenbalg et al., 2013).

The goal of the present study was to investigate the compositional changes of MEVs when isolated from *Mtb* growing under the host-related condition of Fe starvation and test whether immunogenicity changes can improve their biomarker capacity in a new cohort of serum samples from 90 individuals, including 30 TB patients, 30 latent TB infected individuals (LTBI), and 30 healthy volunteers.

## Results

### *Mycobacterium tuberculosis* Modifies the Magnitude and Composition of Mycobacterial Extracellular Vesicle Production When Growing Under Iron Starvation

We have previously shown that *Mtb* and other mycobacteria species produce EVs when cultured in minimal medium (MM) supplemented with Fe (Prados-Rosales et al., 2011). Compositional analysis of High Fe MEVs indicated enrichment in lipoproteins LpqH, LppX, or LprG (Prados-Rosales et al., 2011; Lee et al., 2015); and lipid profile analysis suggested that they originated from the bacterial cell membrane since polar lipids were the most abundant species in High Fe MEVs. Moreover, we have also shown that Fe starvation is a condition that makes *Mtb* overproduce MEVs (Prados-Rosales et al., 2014b). Importantly, iron starvation is a state that induces slow proliferation, suggesting that vesiculation is not strictly linked to the growth rate. Overall, those results showed that the magnitude of the vesiculation process and the composition of MEVs can be altered when *Mtb* faces host-related culturing conditions. However, a comprehensive differential proteomic analysis between High and Low Fe MEVs has never been performed.

**FIGURE 1:**
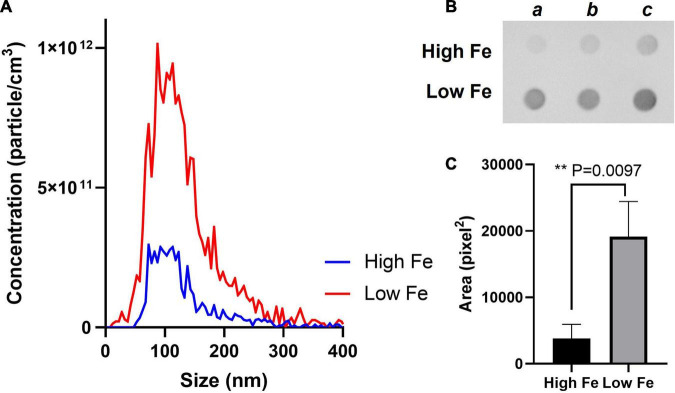
Fe starvation induces vesicle production in *Mtb*. **(A)** MEVs purified from High and Low Fe cultures using sucrose gradient centrifugation and submitted to nanoparticle tracking analysis using ZetaView. Graph shows size distribution (nm) and corresponding to particle concentration (particle cm^– 3^). **(B)** Dot blot analysis of the indicated MEV preparation from three independent cultures (a, b, c) using a specific antibody against MEVs. **(C)** The area (pixel^2^) of the spots calculated in ImageJ as previously described (Ziegenbalg et al., 2013). Graph shows mean values and standard deviation. Results are considered to be statistically significant when *P* < 0.05 according to an unpaired two-tailed *t*-test.

**FIGURE 2:**
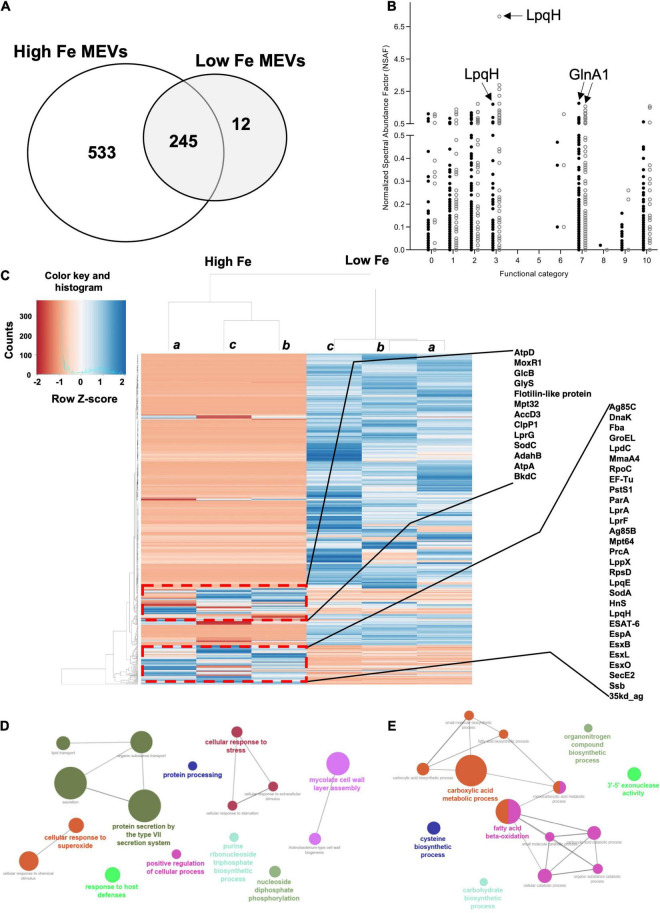
Label-free MS analysis of isolated MEVs. **(A)** Venn diagram showing the total number of proteins identified for both High and Low Fe conditions, distributed between unique and shared proteins for each condition. Analysis was run using Venny 2.0.2 (Oliveros, 2007). **(B)** Distribution of identified MEV-associated proteins in different functional categories for both High Fe (filled circles) and Low Fe (empty circles) MEVs. Functional categories: Virulence, detoxification, adaption (0), lipid metabolism (1), information pathways (2), cell wall and cell processes (3), stable RNAs (4), insertion seqs and phages (5), PE/PPE (6), intermediary metabolism and respiration (7), unknown (8), regulatory proteins (9), conserved hypotheticals (10). Arrows indicate NSAF values of certain MEV proteins such as LpqH or GlnA1 as bonafide MEV markers. **(C)** Heat map showing the expression levels of both Low Fe and High Fe MEV proteins. Samples a, b and c represent independent biological replicates. The blue color indicates up-regulation of proteins, whereas down-regulation is marked in red. Several proteins of two clusters of upregulation in the Low Fe MEV dataset are highlighted. Gene Ontology (GO) analysis of upregulated **(D)** and downregulated **(E)** Low Fe MEV-associated proteins.

We, therefore, proceeded to grow *Mtb* in High and Low Fe conditions and analyzed vesiculation. We confirmed that Fe starvation enhances vesiculogenesis as measured by nanoparticle tracking analysis (NTA) (Figure 1A). Although the average mean diameter of MEVs in High and Low Fe was similar, the concentration of purified MEV from Low Fe condition was fourfold higher than that of High Fe condition (Figure 1A). Alternatively, a specific antibody against MEVs (Rath et al., 2013; Prados-Rosales et al., 2014b) was used to determine MEV production by dot-blot, which indicated Fe starvation induces a statistically significant fivefold increase in MEV production relative to Fe sufficiency conditions (Figures 1B,C). Further, we isolated MEVs from High and Low Fe conditions and determined protein composition by label-free high throughput liquid chromatography-mass spectrometry analysis (LC-MS) (Figure 2 and Supplementary Table 1). We could unequivocally identify 778 proteins in High Fe MEVs and 257 proteins in the subset of Low Fe MEVs, indicating a dramatic reduction in the number of proteins incorporated in MEVs upon Fe starvation (Figure 2A). Despite such reduction in MEV proteins in the Low Fe condition, similarities between both types of MEVs were observed. First, identified proteins appeared to be equally distributed according to functional category between both types of MEVs (Figure 2B). Furthermore, in both High and Low Fe MEVs we could not identify any protein linked to “stable RNAs” or “insertion sequences and phages” functional categories. Moreover, most Low Fe MEV-associated proteins were present in High Fe MEVs and only 12 proteins appeared as unique in MEVs isolated in Fe starvation condition (Figure 2A). Those 12 unique Low Fe-related MEV proteins were mostly low abundant hypothetical proteins without known function. Only the ATP binding subunit ClpC1 and the membrane-bound protease FtsH appear to be well represented in Low Fe MEVs with Normalized Spectral Abundance Factor (NSAF) values of 0.22 and 0.09, respectively (Supplementary Table 1). When looking at the most abundant proteins in each functional category we could notice that they were mostly the same in both conditions (Table 1). Proteins like GlnA1 (NSAF 1.76 High Fe; NSAF 1.57 Low Fe) or LpqH (NSAF 1.71 High Fe; NSAF 7.13 Low Fe) were detected as the most abundant ones in High and Low Fe MEVs, respectively. Of note, LpqH was between 5 to 7-times more abundant in Low Fe than in High Fe MEVs. Nevertheless, the fact that both LpqH and GlnA1 are present in MEVs linked to such divergent conditions, suggests that they may represent *bonafide* MEV markers. These results indicate that the enhanced vesiculation observed in *Mtb* upon Fe starvation is accompanied by a reduction in the number of proteins present in MEVs. In addition, Low Fe MEVs proteins are not unique relative to High Fe MEVs.

**TABLE 1:** Most abundant proteins in High Fe and Low Fe MEVs.

Functional category	High Fe MEVs				Low Fe MEVs			
	# detected proteins	most abundant proteins	Protein name	NSAF	# detected proteins	most abundant proteins	Protein name	NSAF
								
Virulence and detoxification	30	Rv1908c	KatG	1.11	11	Rv1908c	KatG	1.09
		Rv0798c	Cfp29	0.82		Rv0798c	Cfp29	1.07
		Rv2031c	HspX	0.66		Rv0440	GroEL2	0.96
		Rv2740	EphG	0.43		Rv0350	DnaK	0.55
		Rv0350	DnaK	0.32		Rv0432	SodC	0.39
Lipid metabolism	110	Rv0242c	FabG4	0.83	35	Rv0242c	FabG4	1.39
		Rv2831	EchA16	0.59		Rv2244	AcpM	1.2
		Rv0824c	DesA1	0.56		Rv2831	EchA16	1.08
		Rv3800c	Pks13	0.54		Rv0675	EchA5	1.07
		Rv1070c	EchA8	0.51		Rv0642c	MmaA4	0.72
Information pathways	93	Rv0723	RpIO	1.18	41	Rv0054	Ssb	1.73
		Rv0054	Ssb	1.06		Rv0716	RpIE	1.23
		Rv2904c	RpIS	1		Rv0714	RpIN	1.21
		Rv0714	RpIN	0.86		Rv0704	RpIB	0.85
		Rv0716	RpIE	0.84		Rv0701	RpIC	0.85
Cell wall and cell processes	89	Rv3763	LpqH	1.71	44	Rv3763	LpqH	7.13
		Rv3875	EsxA	0.89		Rv3875	EsxA	2.89
		Rv2346c	EsxO	0.61		Rv3616c	EspA	2.64
		Rv1793	EsxN	0.55		Rv3874	EsxB	2.23
		Rv1198	EsxL	0.5		Rv0379	SecE2	1.74
PE/PPE	3	Rv3478	PPE60	0.47	3	Rv3478	PPE60	1.09
		Rv2430c	PPE41	0.37		Rv2430c	PPE41	0.37
		Rv1196	PPE18	0.1		Rv1196	PPE18	0.1
Intermediary metabolism	301	Rv2220	GlnA1	1.76	90	Rv2220	GlnA1	1.57
		Rv2215	DlaT	0.88		Rv1872c	LldD2	1.33
		Rv1023	Eno	0.81		Rv3841	BfrB	1.32
		Rv3841	BfrB	0.75		Rv1327c	GlgE	1.16
		Rv3248c	SahH	0.74		Rv1133c	MetE	1.1
Unknown	1	Rv2818c	Hypothetical protein	0.02	0			
Regulatory proteins	19	Rv1479	MoxR1	0.16	2	Rv1479	MoxR1	0.26
		Rv3295	probably TetR-family	0.11		Rv0144	possibly TetR-family	0.22
		Rv0144	possibly TetR-family	0.11				
		Rv1267c	EmbR	0.1				
		Rv3692	MoxR2	0.08				
Conserved hypotheticals	132	Rv3127	Conserved protein	0.62	31	Rv2744c	Conserved 35 kDa alanine rich protein	1.58
		Rv3131	Conserved protein	0.45		Rv0831c	Conserved protein	1.49
		Rv0831c	Conserved protein	0.44		Rv3127	Conserved protein	0.58
		Rv2744c	Conserved 35 kDa alanine rich protein	0.44		Rv3747	Conserved protein	0.37
		Rv2030c	Conserved protein	0.44		Rv3205c	Conserved protein	0.35

An in-depth protein analysis between both datasets indicated that most proteins were less abundant in Low Fe MEVs relative to the High Fe condition (Figure 2C). Unsupervised clustering analysis of the identified proteins revealed two groups of proteins with higher abundance in Low Fe MEVs, corresponding to lipoproteins, proteins related to the ESX-1 secretion system, and some highly immunogenic proteins such as Ag85bc (Figure 2C). We also observed some proteins related to mycolic acid synthesis. In fact, when running gene ontology (GO) analysis of the Low Fe MEV proteins we could confirm that upregulated proteins were restricted to biological processes such as protein secretion and Type VII secretion system, cellular response to stress to superoxide, mycolic acid synthesis, and nucleoside biosynthesis (Figure 2D). On the other hand, Low Fe MEV downregulated proteins belonged mostly to cellular processes such as beta-oxidation of lipids or metabolism of carboxylic acid (Figure 2E). These results indicate that besides carrying less protein load, Low Fe MEVs incorporate specific proteins related to cell envelope-related processes in higher abundance relative to High Fe MEVs.

### Immunogenicity of High Fe and Low Fe Mycobacterial Extracellular Vesicles

**FIGURE 3:**
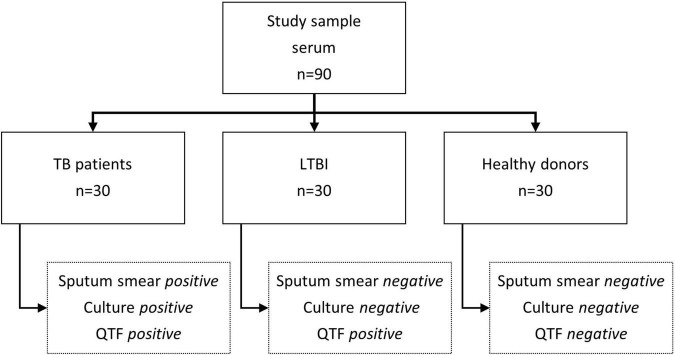
Schematic representation of the characteristics of the serum samples used in the study. TB, tuberculosis, LTBI, Latent tuberculosis infection, QTF, *QuantiFERON*-*TB Gold Plus.*

To expand the catalog of TB biomarkers, we moved to test the immunogenicity of both High and Low Fe MEVs against a different cohort of 90 serum samples, including 30 TB, 30 LTBI individuals, and 30 healthy controls (Figure 3). To achieve this, we first separated equivalent amounts (based on NTA measurements) of High Fe and Low Fe MEVs by SDS-PAGE in a range (10–250 kDa) and submitted them to immunoblot against serum samples from different groups.

#### Immunogenicity of High Fe Mycobacterial Extracellular Vesicles

**FIGURE 4:**
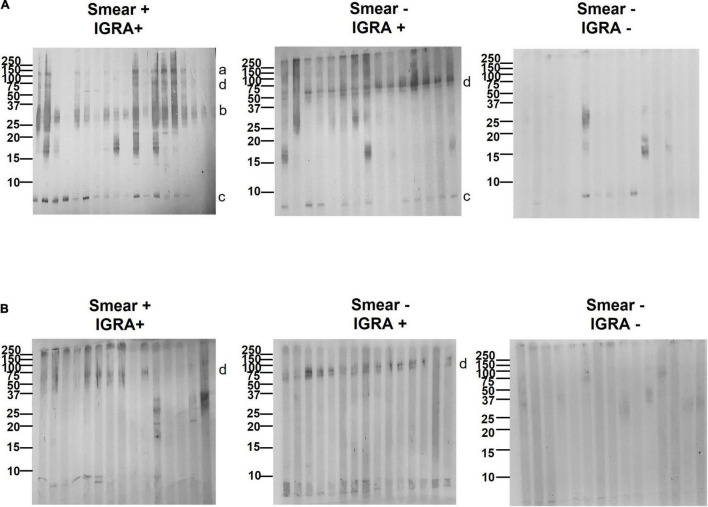
Serological reactivity pattern of High Fe and Low Fe MEVs. Immunoblots of 15–18 representative serum samples from smear+, IGRA+ (TB), smear-, IGRA+ (Latent TB), and smear-, IGRA- (healthy) individuals against High Fe **(A)** and Low Fe **(B)** MEVs. MEVs were separated by SDS-PAGE and blotted with the serum samples. Membranes were further probed with an anti-human IgG secondary antibody conjugated with horseradish peroxidase (HRP) and developed with 3,3’,5,5’-Tetramethylbenzidin (TMB). Reactive MEV-associated protein bands a, b, c, and d are indicated next to the blots. For clarity, only a subset of standard marker bands (250–10 kDa, Bio-Rad, Hercules, CA, United States) is indicated.

We proceeded to analyze the immunoblot profiles of the different sets of EVs against three types of serum samples: (a) smear+, culture+, IGRA+ (TB); (b) smear-, culture-, IGRA+ (LTBI); and (c) smear-, culture-, IGRA- (healthy volunteers). The results showed a change in the number of reactive bands according to the disease status (Figure 4). We observed more reactive bands in samples from individuals with active TB than in LTBI and healthy individuals, which is consistent with our previous study (Ziegenbalg et al., 2013) and other serological studies (Steingart et al., 2009). When looking at the band pattern, we could observe reactivity against a band > 100 kDa (band a) in 23/30 of TB samples, a smeared band < 37 kDa (band b) in 28/30 TB samples, a band < 10 kDa (band c) in 28/30 TB samples, and a double band < 50–60 kDa (band d) in 10/30 TB samples (Figure 4). Interestingly, no reactivity against band a was observed in any of the LTBI samples analyzed (0/30). However, we could detect reactivity against band d in 28/30 LTBI samples and reactivity against band c was observed in 20/30 LTBI samples. Like band a, reactivity against band b was absent in most of the LTBI samples (20/30) (Figure 4). Finally, when looking at samples from healthy volunteers almost no reactivity was detected for bands observed in the other set of samples (Figure 4). These results indicate antibody (Ab) reactivity to High Fe MEV antigens in a higher proportion of smear-positive (TB) compared to smear-negative (LTBI) patients and identify additional MEV proteins with the capacity to discriminate TB from non-TB individuals by serology.

#### Immunogenicity of Low Fe Mycobacterial Extracellular Vesicles

We next moved to investigate the immunogenicity of Low Fe MEV for the first time. When checking the immunogenicity of Low Fe MEVs against smear+, IGRA+ samples, we noticed a dramatic reduction in the number of bands detected relative to High Fe MEVs (Figure 4). No reactivity was observed for bands a, b, or c. Regarding band d, it could only be detected in 12/30 TB samples. However, when immunoblots were analyzed using smear-, IGRA+ samples (LTBI), band d could be observed in 26/30 samples (Figure 4). The preferential recognition of band d for latent TB serum samples seems to be conserved between High and Low Fe MEVs. Further, no reactivity against low Fe MEVs was detected in samples from healthy volunteers.

Overall, these results confirm that MEVs are a source of TB biomarkers with the capacity to discriminate between TB disease status by serology. In addition, evidence was provided for the recognition of additional MEV antigens with the potential to discriminate TB from non-TB individuals, as well as active TB from latent TB ones. On the other hand, Low Fe MEVs do not seem to carry additional biomarkers in comparison to High Fe MEVs.

### Performance of Disease Biomarkers

**FIGURE 5:**
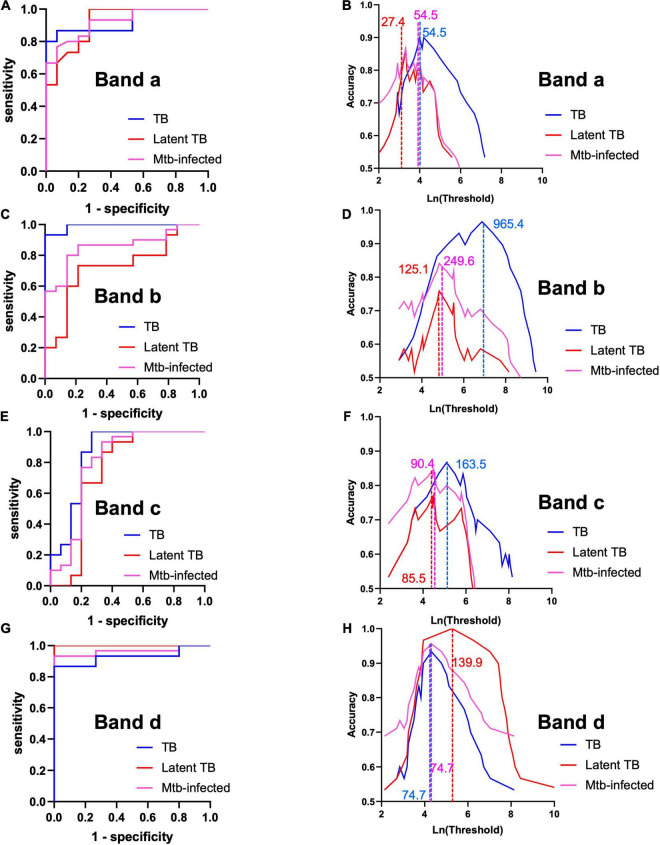
Perfomance analysis of each reactive band. **(A,C,E,G)** Receiver-operating characteristic (ROC) curves for each band (Table 2). **(B,D,F,H)** Optimal threshold analysis based on accuracy measurements for each band and sample type. Lines and numbers indicate the optimal threshold (pixel^2^) of the indicated band for each sample type. Analyses were performed for bands a, b, and c from High Fe MEV blots, while band d was analyzed from Low Fe MEVs blots.

Having established the similarities and differences between each set of MEVs regarding reactivity against serum samples from TB cases and controls, we proceeded to determine the marker which best discriminate between sample type. Analyses were performed for bands a, b, and c from High Fe MEV blots, while band d was analyzed from Low Fe MEVs blots. At first glance, the immunogenicity analysis seemed to differentiate between TB+ (smear+, IGRA+ and smear-, IGRA+) and TB- (smear-, IGRA-) samples, since no reactivity was observed in samples from healthy volunteers for most of the selected bands. All selected bands were somewhat correlated [Spearman’s coefficients range from 0.69 (*p*-value < 0.001)] for bands A vs. B to 0.30 (*p*-value 0.047) for bands c vs. d (Supplementary Table 2). Band areas were consistently higher for latent and active TB cases than for healthy volunteers (Supplementary Table 3). Notably, the area for band d, contrary to other bands’ areas, was higher for latent [median (interquartile range) 2441.15 pixel^2^ (1758.41–3237.36)] rather than for active cases [370.21 pixel^2^ (150.56–750.02)] (Supplementary Table 3). We next run receiver-operating characteristic (ROC) analyses (Figures 5A,C,E,G and Table 2) and found that band a, band b and band d have optimal overall performance for the distinction between TB cases and controls. Band a seems to reliably identify smear+ IGRA+ [AUC 0.924, 95% CI (0.82–1)] (Figure 5A and Table 2) latent [0.918 (0.82–1)] and all TB cases (*Mtb*-infected) [0.921 (0.85–1)]. Band b showed a great power to identify smear+ TB+ individuals [0.99 (0.97–1)] but poorer capacity for latent [0.72 (0.52–0.92)] and all TB cases (*Mtb*-infected) [0.85 (0.74–0.97)] (Figure 5C and Table 2). Of note, band d showed the strongest capacity to identify latent TB cases [AUC 1.0 (1.0–1.0)] compared to the other bands (Figure 5G and Table 2). Conversely, band c showed lower AUC values relative to the other bands [AUC 0.86 (0.71–1), 0.74 (0.54–0.95), 0.80 (0.63–0.97) for TB+, latent and all TB cases (*Mtb*-infected), respectively] (Figure 5E and Table 2). Statistically significant differences between AUC values were only found for bands b vs. d (DeLong test *p*-value, 0.009), and bands c vs. d (*p*-value, 0.013) when discriminating between latent TB cases and healthy controls (Supplementary Table 4). We assessed the performance of the different markers at the optimal threshold which provided maximal accuracy (Figures 5B,D,F,H and Table 2). Unsurprisingly, we found that most bands have a specific optimal cut-off point according to disease status. For instance, a cut-off point of 54 pixel^2^ for band a gives 0.90 (95% CI, 0.8–1) and 0.82 (0.71–0.93) accuracy for the detection of smear+ and TB+ cases, respectively, while a cut-off point of 27.4 pixel^2^ gives 0.86 (95% CI 0.73–0.97) accuracy for the identification of latent TB cases (Figure 5B). These results suggest that band a preferentially detects active TB individuals. This performance was very similar for band b by setting the cut-off point at 965.4 pixel^2^ when checking for smear+ TB+ cases [accuracy 0.97 (0.9–1)] (Figure 5D). Importantly, a threshold of 193.9 pixel^2^ for band d gives the highest accuracy [1 (1 -1)] for the detection of latent TB cases, while a cut-off of 74.75 pixel^2^ have 93% (0.83–1) for active TB ones. Thus, a threshold of 74.75 pixel^2^ could allow the detection of both smear+ and smear- cases (Figure 5H). Taken together, these results show that MEVs carry proteins with great biomarker potential for the distinction between TB+ individuals and healthy controls, as well as smear+ TB+ from smear- TB+ cases.

**TABLE 2:** Performance metrics of MEV reactive bands.

	band a			band b			band c			band d		
	TB	Latent TB	*Mtb*-infected	TB	Latent TB	*Mtb*-infected	TB	Latent TB	*Mtb*-infected	TB	Latent TB	*Mtb*-infected
AUC	0.924	0.918	0.921	0.99	0.72	0.85	0.86	0.74	0.8	0.93	1	0.96
95% CI	0.82–1	0.82–1	0.85–1	0.97–1	0.52–0.92	0.74–0.97	0.71 –1	0.54–0.95	0.63–0.97	0.82–1	1–1	0.91–1
Accuracy*	0.9	0.86	0.82	0.965	0.758	0.818	0.866	0.766	0.844	0.933	1	0.955
95% CI	0.8–1	0.73–0.97	0.71–0.93	0.9–1	0.59–0.9	0.7–0.9	0.7–0.97	0.6–0.9	0.75–0.93	0.83–1	1–1	0.89–1
Sensitivity*	0.87	1	0.77	0.93	0.73	0.8	1	0.93	0.93	0.87	1	0.93
95% CI	0.67–1	1–1	0.6–0.9	0.8–1	0.47–0.93	0.67–0.93	1–1	0.8–1	0.83–1	0.67–1	1–1	0.63–1
Specificity*	0.93	0.73	0.93	1	0.79	0.86	0.73	0.6	0.67	1	1	0.96
95% CI	0.8–1	0.47–0.93	0.8–1	1–1	0.57–1	0.64–1	0.47–0.93	0.33–0.8	0.4–0.87	1–1	1–1	0.89–1

AUC, Area under curve; CI, confidence interval. *Assessed at the highest accuracy.

## Discussion

The lack of correlates of protection, diagnostic or prognosis for tuberculosis (TB) represents one of the main hurdles at designing strategies to tackle the disease. In addition, there is also a need for biomarkers to speed up the assessment of TB treatment. TB serology can be an attractive option for resource-limiting settings for TB diagnosis in the form of a POC test. In fact, researchers have tried to develop serology tests aimed at differentiating TB from LTBI for decades. Those commercialized are variable in sensitivity (10–90%) and specificity (47–100%) and this depends on antigen included, subjects, or site, among other factors (Steingart et al., 2007; Achkar et al., 2011). Besides the intrinsic heterogeneity of antibody (Ab) responses in TB individuals (Kunnath-Velayudhan et al., 2010), most assays are based on antigens that elicit Ab responses in individuals with advanced TB. In addition, early TB stages are linked to a low bacterial burden with the concomitant reduction in Ab titers. Interestingly, HIV-infected TB patients seem to develop Ab responses to a smaller repertoire of *Mtb* antigens compared to HIV-uninfected TB patients (Sartain et al., 2006). Although specificity has also been shown to be insufficient in TB serology tests, mostly due to the potential contribution of responses due to non-tuberculous mycobacteria or BCG vaccination, some antigens have been shown to specifically trigger TB-specific Ab responses (Hendrickson et al., 2000; Achkar et al., 2010).

Intending to expand the catalog of TB biomarkers, we have studied protein composition changes in MEVs isolated from *Mtb* submitted to a host-related condition such as Fe starvation, relative to Fe sufficiency. When residing intracellularly, *Mtb* faces Fe starvation, a strategy from many hosts that restrict access to Fe upon infection (Weinberg, 1984). Consequently, *Mtb* reduces its proliferation rate and activates a Fe starvation-specific transcriptional program which triggers the synthesis of Fe-binding molecules (mycobactins) (Rodriguez and Smith, 2006). In addition, it is reasonable to assume that *Mtb* also modifies protein composition to adapt to this very stressful and restrictive environment. Indeed, a previous transcriptional study revealed that Fe deficiency induces the expression of genes needed for the subversion of macrophage antimicrobial defense, such as those related to type VII secretion systems, the protein kinase PknG and other virulence-associated factors, including sulfolipid biosynthesis, cholesterol utilization genes, isocitrate lyase, phosphoenolpyruvate carboxykinase, and WhiB6 (Kurthkoti et al., 2017). We have previously shown that another manifestation of the response of *Mtb* to Fe deprivation is the enhancement in MEV production (Prados-Rosales et al., 2014b). We determined by metabolomics that low Fe MEVs load mature Fe-loaded mycobactins, whereas mostly Fe-free mycobactin intermediates are abundant in High Fe MEVs, indicating changes in vesicle cargo (Prados-Rosales et al., 2014b). In addition, we demonstrated growth rescue of mutants unable to grow in Fe-deficiency without mycobactins upon supplementation with low Fe MEVs (Prados-Rosales et al., 2014b), indicating a role for MEVs in pathogenesis.

To fully understand how *Mtb* modifies the whole MEV cargo upon Fe deprivation, we conducted a high throughput proteomic analysis and found changes in the cargo and number of Low-Fe MEV associated proteins relative to High Fe MEVs (Figures 1, 2). Specifically, as previously shown, MEVs are produced in higher quantity in Fe deprivation but *Mtb* seems to load fewer proteins compared to Fe sufficiency. This is probably reflecting the slow replicating phenotype imposed by such stressful condition. In fact, it is known that growth restriction is concomitant to a reduction in protein synthesis (Hu et al., 1998). Interestingly, Low Fe MEVs do not seem to load different proteins than High Fe MEVs but in different quantities. Notably, differential proteomic analysis between both types of MEVs showed that Low Fe MEVs incorporated higher amounts of lipoproteins, and substrates of the ESX-1 Type VII secretion system, as well as other immunogenic proteins such as Ag85bc. The correlation between the increase in abundance of these proteins and the enhanced vesiculation phenomenon in Fe starvation may suggest that these proteins are important for vesicle production in *Mtb*. In fact, lipoprotein LpqH has been previously shown to interact with VirR (Rv0431) a negative regulator of vesicle production (Rath et al., 2013). In addition, the high degree of conservation in the most abundant MEV proteins between such divergent conditions in term of growth and metabolism such as Fe sufficiency and Fe deprivation clearly indicates the establishment of certain MEV proteins such as LpqH or GlnA1 as *bonafide* MEV markers. This information has important implications in diagnostic settings aimed at detecting and/or immunocapturing MEVs in clinical samples.

Having established the compositional changes between High and Low Fe MEVs we continued to test their immunogenicity against serum samples from a cohort of 90 individuals distributed between smear+ TB+, smear- TB+, and healthy controls (Figure 3). We have previously demonstrated that High Fe MEVs are strongly immunogenic in HIV uninfected patients with pulmonary TB (Ziegenbalg et al., 2013). These results indicated that some High Fe MEV-associated proteins elicit serum IgG responses in both smear-positive and smear-negative TB patients but not in TST + BCG vaccinated healthy controls with or without LTBI. A biomarker signature including three MEV proteins of ∼36, 25, and 23 kDa could simultaneously differentiate between TB and not TB states in immunoblots, suggesting great diagnostic potential with high sensitivity and specificity. This study included MEV isolated from *Mtb* growing under Fe sufficiency, a condition that does not resemble what *Mtb* may face when residing in the intracellular environment. Moreover, the size resolution provided by immunoblots in that study was restricted to protein masses between 20 and 250 kDa (Ziegenbalg et al., 2013). Our study did partially reproduce the above results, since we saw robust reactivity for band b (> 37 kDa) in smear+ TB+ samples. Regarding band 23 and 25 kDa proteins, we could see reactivity in 10/30 samples. We suppose that both the cohort geography and the inherent variability of MEV composition (Prados-Rosales et al., 2014a) could explain such differences. However, regarding the latter, we tried to reproduce the exact conditions to isolate MEVs in the former study (Ziegenbalg et al., 2013). Overall, we identified four protein bands that could be preferentially recognized in TB samples over healthy controls. All bands were present in High Fe MEVs. We could not identify additional proteins in Low Fe MEVs relative to High Fe MEVs with biomarker capacity. This was initially unexpected but can be explained by the finding that most of the Low Fe MEV proteome is present in High Fe MEVs (Figure 2). In fact, we observed a strong reduction in reactivity for most of the bands detected in High Fe MEVs with band d being the only Low Fe MEV protein robustly recognized by sera. Such differences can be explained by either a change in antibody specificity according to disease status or by changes in protein abundance within MEVs. Further performance analysis indicated that bands a and b have superior capacity to detect individuals with active TB vs. latent TB, while band d was a suitable marker to detect latent TB individuals. Therefore, Ab responses to proteins a, b, and d enriched in MEVs constitute a novel TB Ab biomarker signature with diagnostic information. Moreover, the fact that reactivity against Low Fe MEVs is restricted to band d and in the context of LTBI, we can argue that Low Fe MEV could be useful to discriminate between both TB from not-TB individuals and TB from LTBI individuals.

In conclusion, this study provides valuable information for future developments in TB diagnostic with a focus on MEVs: (i) the identification of *bonafide* MEV markers which could be used for direct diagnosis; (ii) a novel biomarker signature of three MEV-proteins with potential in the indirect diagnostic of the disease. In any case, future studies are needed to establish the feasibility of MEVs as a platform to improve TB diagnostics.

## Materials and Methods

### Bacteria and Culture

*Mtb* H37Rv (ATCC) was initially grown in Middlebrook 7H9 medium (7H9) supplemented with 10% (v/v) OADC enrichment (Becton Dickinson Microbiology Systems, Spark, MD, United States), 0.5% (v/v) glycerol and with or without Tyloxapol 0.05% (v/v; Sigma) prior to inoculation in a MM consisting of KH_2_PO_4_ 1 g/l, Na_2_HPO_4_ 2.5 g/l, asparagine 0.5 g/l, ferric ammonium citrate 50 mg/l, CaCl_2_ 0.5 mg/l, ZnSO_4_ 0.1 mg/l, with or without Tyloxapol 0.05% (v/v), containing 0.1% (v/v) glycerol, pH 7.0. Cultures were grown in 7H9 at 37°C until they reached an OD of 0.6 and then washed in in phosphate-buffered saline (PBS) + 0.05% Tyloxapol and transferred to MM. Low iron minimal medium (LIMM) was used for culture under iron-controlled conditions. To remove metal ions MM was treated with 5% Chelex-100 (Bio-Rad, Hercules, CA, United States) for 24 h, at 4°C with gentle agitation. Chelex-100 was removed by filtration through 0.22 μm filter (Millipore, Burlington, MA, United States) and the medium was supplemented with sterile ZnCl_2_ 0.5 mg/l, of MnSO_4_ 0.1 mg/l, and of MgSO_4_ 400 mg/l. The amount of residual Fe in this medium, determined by atomic absorption spectroscopy is ∼1 μM. High iron MM (MM) was prepared by supplementing LIMM with 50 μM FeCl_3_.

### Isolation of Mycobacterial Extracellular Vesicles

Mycobacterial extracellular vesicles were prepared and purified as previously described (Prados-Rosales et al., 2011). Briefly, the culture filtrate of 1 L cultures of *Mtb* grown in MM and LIMM without detergent for 14 days was processed for vesicle isolation by differential centrifugation. In parallel a 2 ml culture in MM supplemented with 0.05% tyloxapol to disperse bacterial clumps were used to determine viability by plating culture dilutions onto 7H10 agar plates and enumerating colony-forming unit (CFU) at the time of culture filtrate collection. The membranous pellet containing vesicles, protein aggregates and capsule polysaccharides obtained after ultracentrifugation of the culture filtrate at 100,000 × *g* for 2 h at 4°C, was resuspended in 1 ml sterile PBS and overlaid with a series of Optiprep gradient layers with concentrations ranging from 45 to 20% (w/v). The gradients were centrifuged at 100,000 × *g* for 16 h. At the end 1 ml fractions were removed from the top, diluted to 20 ml with PBS and purified vesicles recovered by sedimentation at 100,000 × g for 1 h. Vesicle pellets were suspended in PBS before analysis. Protein concentration was measured by Bradford assay (Bio-Rad, Hercules, CA, United States) according to manufacturer instructions.

### Nanoparticle Tracking Analysis

Nanoparticle tracking analysis was conducted using ZetaView (Particle Metrix, WInning am Ammersee, Germany). Instrument calibration was performed prior to EV analysis using 102 nm polystyrene beads (Thermo Fisher Scientific, Walthman, MA, United States), according to manufacturer instructions. Measurements were performed using a 405 nm 68 mW laser and CMOS camera by scanning 11 cell positions and capturing 60 frames per position at 25^°^C with camera sensitivity 85, shutter speed 100, autofocus and automatic scattering intensity. Samples were diluted in pre-filtered PBS to approximately 10^6^–10^7^ particles⋅ml^–1^ in Millipore DI water. Analysis was performed using ZetaView Software version 8.05.12 SP1 with a minimum brightness of 30, maximum brightness of 255, minimum area of 5, maximum area of 1,000, and s minimum trace length 15. Triplicate videos of each sample were taken in light scatter mode. Particle size and concentration were analyzed using the built-in EMV protocol and plotted using Prism software, version 8.0.1 (GraphPad Inc., San Diego, CA, United States).

### Label-Free Mass Spectrometry Analysis

Mycobacterial extracellular vesicle-associated proteins were submitted to LC-MS label-free analysis using a hybrid trapped ion mobility spectrometry–quadrupole time of flight mass spectrometer (timsTOF Pro with PASEF, Bruker Daltonics, Billerica, MA, United States) coupled online to a nanoElute liquid chromatograph (Bruker, Billerica, MA, United States). Samples were incubated and digested following the filter-aided sample preparation (FASP) protocol (Meier et al., 2018). Trypsin was added to a trypsin:protein ratio of 1:50, and the mixture was incubated overnight at 37°C, dried out in a RVC2 25 speedvac concentrator (Christ), and resuspended in 0.1% FA. Peptides were desalted and resuspended in 0.1% FA using C18 stage tips (Millipore). Digested sample (200 ng) was loaded in a 15 cm Bruker nanoelute FIFTEEN C18 analytical column (Bruker) and resolved at 400 nL/min with a 30 min gradient (gradient from 3 to 40% B, where A: water with 0.1% FA and B: acetonitrile with 0.1% FA). Data was acquired using the standard DDA PASEF-standard_1.1sec_cycletime method, that scans between 100–1,700 m/z, and 0.60–1.60 v.s/cm^2^ using a ramp time of 100 ms. The column was heated to 50°C using an oven. Protein identification and quantification were carried out using Peaks software using default settings (Bioinformatics solutions). Searches were carried out against a database consisting of *Mtb* H37Rv protein entries (NCBI), with precursor and fragment tolerances of 20 ppm and 0.05 Da. Only proteins identified with at least two peptides at FDR < 1% were considered for further analysis. Quantification of protein abundance was performed by calculating the NSAF (Bubis et al., 2017). The NSAF is the number of spectral counts (SpC, the total number of MS/MS spectra) detected for a specific protein, k, divided by the protein’s length (L), divided by the sum of SpC/L for all N proteins in the experiment (Zybailov et al., 2006). NSAF values were calculated from three biological replicates of the indicated conditions. Alternatively, identified proteins were classified by functional category according to the Mycobrowser database (Kapopoulou et al., 2011). Heatmaps were plotted *via* heatmap.2 function from the gplots R package,^1^ using a regularized log transformation (RLB) on NSAF values to enhance the graphical representation of the data. The histogram in the heatmap indicates normalized protein abundance (*z*-score) represented by a color scale. The white color represents a *z*-score of 0 and gradients of red and blue increase toward the z-scores around −2/+2, respectively. Enrichment analysis of GO terms was performed using the ClueGO package (Bindea et al., 2009) using a Benjamini-Hochberg method and *p*-value less than equals to 0.05, keeping predefined kappa threshold value.

### Serum Samples

A total of 90 sera was obtained from 90 patients at the Vall d’Hebron Hospital of Barcelona during 2017. The sera were selected based on the results of the smear microscopy, mycobacterial culture, and QuantiFERON-TB Gold Plus (QTF). The sera were distributed as follows: 30 sera from TB patients (smear-positive, culture positive for *Mycobacterium tuberculosis* and QTF positive), 30 sera from LTBI patients (smear negative, culture negative for *M. tuberculosis* and QTF positive), and 30 sera from healthy people (smear-negative, culture-negative for *M. tuberculosis* and QTF negative). The current study was approved by the Ethical Committee of Vall d’Hebron Hospital of Barcelona. The informed consent of the patients was not required since the sera obtained for the study came from remnants of samples that were used in the routine hospital care and no data related to them has been used.

### Immunoblot

Antibody response profiles to MEV-associated proteins were analyzed by immunoblotting. A solution with 30 μg of MEVs was separated by SDS-PAGE and transferred to a nitrocellulose membrane before blocking in 5% skimmed milk in PBS + 0.01% Tween 20 (PBS-T) for 1 h. Serum samples diluted at 1:100 in blocking buffer were incubated at 4°C overnight using the multi-channel blotting system Surf-Blot 7.5 (Idea Scientific, MN, United States). The membranes were washed in PBS-T and incubated for 1 h an HRP-conjugated goat antibody to human immunoglobulin G (Sigma) and the bands were visualized using 1-step™ ultra TMB-blotting substrate solution (Thermo). Alternatively, to perform Western dot blot, 2 μl of 1:200 dilutions of purified EVs were spotted onto a nitrocellulose membrane (GE Healthcare, Marlborough, MA, United States) and incubated with anti-MEV polyclonal murine serum as primary antibody. Antigen-antibody complexes were detected using anti-mouse IgG and the ECL prime Western Blotting Detection chemiluminescent kit (GE Healthcare, Marlborough, MA, United States). The signal was visualized in a Chemidoc MP imaging system (Bio-Rad, Hercules, CA, United States).

### Image Analysis

Band and spot intensities were analyzed by densitometry using ImageJ (Schneider et al., 2012). Backgrounds were determined for each lane individually and set as a straight line at the lowest point of the histological profile of the lane.

### Statistics (ROC and Accuracy Analyses)

Band areas were summarized using median (interquartile range), and their correlations were evaluated using Spearman’s rank correlation test. The overall performance of each reactive band was assessed by estimating the area under the ROC (AUC) and its 95% confidence interval (95% CI). Differences between AUC values were evaluated using DeLong’s test. The optimal band area cut-off for the discrimination between cases [active TB, latent TB, and All TB (*Mtb*-infected)] and healthy controls was determined as the point which maximized the overall classification accuracy (i.e., the total correct classification rate of each TB status). Analyses were performed using the “pROC” package (version 1.18.0) (Robin et al., 2011) in R software (version 4.1.2 R Core Team, 2021); statistical significance was set at a *p*-value < 0.05 (two-tailed). Graphics were generated using Prism software, version 8.0.1 (GraphPad Inc., CA, United States).

## Data Availability Statement

The datasets presented in this study can be found in online repositories. Proteomics data have been deposited on the ProteomeXchange Consortium database via the PRIDE partner repository with the dataset identifier PXD033170.

## Ethics Statement

The current study was approved by the Ethical Committee of Vall d’Hebron Hospital of Barcelona. The informed consent of the patients was not required since the sera obtained for the study came from remnants of samples that were used in the routine care of the hospital and no data related to them has been used. The patients/participants provided their written informed consent to participate in this study.

## Author Contributions

SG and RP-R designed and coordinated the study and wrote the manuscript. SS, LR, SA, MD-V, VS, LL, JS-M, MA, FE, JL, and MG performed experiments and analyzed data. MT coordinated collection of clinical samples. All authors read the manuscript and concur with the current submission.

## Conflict of Interest

The authors declare that the research was conducted in the absence of any commercial or financial relationships that could be construed as a potential conflict of interest.

## Publisher’s Note

All claims expressed in this article are solely those of the authors and do not necessarily represent those of their affiliated organizations, or those of the publisher, the editors and the reviewers. Any product that may be evaluated in this article, or claim that may be made by its manufacturer, is not guaranteed or endorsed by the publisher.

## Footnotes

1
https://CRAN.R-project.org/package=gplots
